# Xenogenomics: Genomic Bioprospecting in Indigenous and Exotic Plants Through EST Discovery, cDNA Microarray-Based Expression Profiling and Functional Genomics

**DOI:** 10.1002/cfg.475

**Published:** 2005-06

**Authors:** Ulrik P. John, German C. Spangenberg

**Affiliations:** Plant Biotechnology Centre Australian Centre for Plant Functional Genomics Victorian Centre for Plant Functional Genomics Primary Industries Research Victoria, La Trobe University Victoria Bundoora 3086 Australia

## Abstract

To date, the overwhelming majority of genomics programs in plants have been directed at model or crop plant species, meaning that very little of the naturally occurring sequence diversity found in plants is available for characterization and exploitation. In contrast, ‘xenogenomics’ refers to the discovery and functional analysis of novel genes and alleles from indigenous and exotic species, permitting bioprospecting of biodiversity using high-throughput genomics experimental approaches. Such a program has been initiated to bioprospect for genetic determinants of abiotic stress tolerance in indigenous Australian flora and native Antarctic plants. Uniquely adapted Poaceae and Fabaceae species with enhanced tolerance to salt, drought, elevated soil aluminium concentration, and freezing stress have been identified, based primarily on their eco-physiology, and have been subjected to structural and functional genomics analyses. For each species, EST collections have been derived from plants subjected to appropriate abiotic stresses. Transcript profiling with spotted unigene cDNA micro-arrays has been used to identify genes that are transcriptionally modulated in response to abiotic stress. Candidate genes identified on the basis of sequence annotation or transcript profiling have been assayed *in planta* and other *in vivo* systems for their capacity to confer novel phenotypes. Comparative genomics analysis of novel genes and alleles identified in the xenogenomics target plant species has subsequently been undertaken with reference to key model and crop plants.


**Comparative and Functional Genomics**
*Comp Funct Genom* 2005; **6**: 230–235.
Published online in Wiley InterScience (www.interscience.wiley.com). **DOI:** 10.1002/cfg.475

**Conference Paper**

### Xenogenomics: genomic bioprospecting in indigenous and exotic plants through EST discovery, cDNA microarray-based expression profiling and functional genomics

Ulrik P. John and German C. Spangenberg*
*Plant Biotechnology Centre, Australian Centre for Plant Functional Genomics, Victorian Centre for Plant Functional Genomics, Primary Industries Research Victoria, La Trobe University, Victoria 3086, Australia*

**Correspondence to:
German C. Spangenberg, Plant Biotechnology Centre, Primary Industries Research Victoria, La Trobe University, Bundoora, Victoria 3086, Australia.
E-mail: German.Spangenberg@dpi.vic.gov.au*

### Abstract

**To date, the overwhelming majority of genomics programs in plants have been directed at model or crop plant species, meaning that very little of the naturally occurring sequence diversity found in plants is available for characterization and exploitation. In contrast, 'xenogenomics' refers to the discovery and functional analysis of novel genes and alleles from indigenous and exotic species, permitting bioprospecting of biodiversity using high-throughput genomics experimental approaches. Such a program has been initiated to bioprospect for genetic determinants of abiotic stress tolerance in indigenous Australian flora and native Antarctic plants. Uniquely adapted Poaceae and Fabaceae species with enhanced tolerance to salt, drought, elevated soil aluminium concentration, and freezing stress have been identified, based primarily on their eco-physiology, and have been subjected to structural and functional genomics analyses. For each species, EST collections have been derived from plants subjected to appropriate abiotic stresses. Transcript profiling with spotted unigene cDNA micro-arrays has been used to identify genes that are transcriptionally modulated in response to abiotic stress. Candidate genes identified on the basis of sequence annotation or transcript profiling have been assayed *in planta* and other *in vivo* systems for their capacity to confer novel phenotypes. Comparative genomics analysis of novel genes and alleles identified in the xenogenomics target plant species has subsequently been undertaken with reference to key model and crop plants.**
**Copyright © 2005 John Wiley & Sons, Ltd.**

Received: 2 February 2005
Accepted: 15 March 2005

**Keywords: biosphere genomics; plant biodiversity; expressed sequence tags (ESTs); microarrays; abiotic stress tolerance; freezing tolerance; ice recrystallization inhibition proteins; antarctic hairgrass**

### Introduction

Global agriculture relies on a very small proportion of the total number of vascular plant species. With one exception (macadamia nut), Australia's plant-derived agricultural economy is based on exotic species that were not initially bred or selected for their ability to thrive under Australian conditions. By contrast, indigenous Australian plants have largely evolved in isolation and are uniquely adapted to a diversity of local environments and to a range of abiotic stresses, including salinity, drought, temperature extremes, nutrient deprivation and acidic soils/aluminium toxicity. Australia is estimated to harbour 10% of the world's total biodiversity, while 85% of its flowering plants are found nowhere else in the world. Native plants are thus a potentially rich resource for the discovery of novel genes and gene variants.

To date, most genomics programs in plants (and other organisms) have been directed at a limited range of species, selected either because

Copyright © 2005 John Wiley & Sons, Ltd.

of their status as model organisms or because of their economic importance. Of the 48 plant species with significant numbers of EST sequences deposited in public databases, the vast majority correspond to model and crop plants [5]. Very few plant species have been targeted for gene discovery on the basis of their enhanced tolerance to abiotic stresses. Notable exceptions include the halophytes *Mesembryanthemum crystallinum* (common ice plant) [3] and the mangrove species *Avicennia marina* [4].

We have coined the term 'xenogenomics' to describe structural and functional genomics specifically targeting non-model and non-crop plants, and we are focusing our efforts on plants with enhanced tolerance to abiotic stresses. In effect, we are bioprospecting for genetic resources in a similar way to that in which screening of biota of different ecosystems for bioactive compounds has yielded the antibiotic erythromycin, the anti-rejection drug cyclosporin A and the anti-proliferative agent taxol.

The xenogenomics program aims to: (a) discover novel genes and alleles determining the molecular basis of abiotic stress tolerance in indigenous Australian grasses and legumes and antarctic grasses that show unique modes of adaptation to stressful environments; (b) engineer coding and regulatory sequences capable of conferring tolerance of, or inducible expression in response to, abiotic stress; and (c) derive model and agronomically important plant genotypes with enhanced tolerance to abiotic stress.

This review provides a brief overview of the initial target species that have been selected for xenogenomic analysis, outlines the principal methodologies that have been applied and describes progress using some of the results that these methodologies have produced to date as examples.

### Xenogenomics target species

Members of the genus *Lachnagrostis* exhibit tolerance to both waterlogging stress and high salinity. The closely related species *L. robusta* (salt-blown grass) and *L. adamsonii* (Adamson's bent grass) are typically found growing in and around seasonally wet, saline depressions in the basalt plains grassland habitat of south-eastern Australia. As a consequence, both species have to cope annually with both submergence and, as summer proceeds, increasing drought and salt stress. *L. robusta* is moderately salt-tolerant, while *L. adamsonii* is highly tolerant, able to thrive in hydroponic media containing 300 mM NaCl.

*Microlaena stipoides* (weeping grass) is a perennial grass that inhabits a wide range of habitats across Australia. It is particularly well adapted to acidic soils and is highly tolerant of the associated elevated levels of potentially toxic $Al^{3+}$ ions. *M. stipoides* has a rapidly induced, highly efficient aluminium exclusion mechanism [2]. Within 2 hours of exposure to 1 mM aluminium at pH 4.0 its roots cease to assimilate aluminium (Figure 1A).

With respect to low temperature and freezing stress — also combined with desiccation stress — we have looked beyond Australia to Antarctica, which is one of the most extreme environments inhabited by higher plants. *Deschampsia antarctica* (Antarctic hair grass) is one of only two vascular plants to have overcome the geographical and environmental impediments to colonization of the Antarctic continent. It is an overwintering species that grows in sheltered locations along the western coast of the Antarctic peninsula. Laboratory studies have demonstrated that *D. antarctica* is truly freezing-tolerant: significant cellular damage only

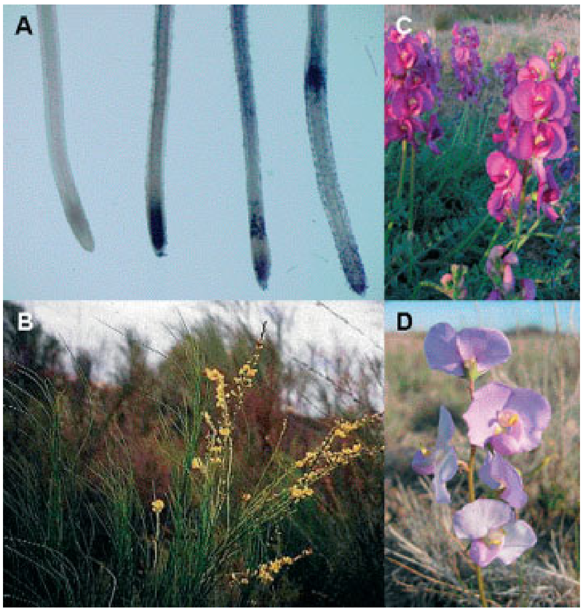

**Figure 1.** Xenogenomics target species. (A) Haematoxylin-stained seedlings of *M. stipoides* exposed to 1 mM $Al^{3+}$ at pH 4.0 for (from left) 0, 1, 6 and 24 h. (B) *Viminaria juncea.* (C) *Swainsona swainsonoides.* (D) *S. procumbens*

Copyright © 2005 John Wiley & Sons, Ltd.

occurs in plants exposed to temperatures substantially below those at which they freeze [1].

Mirroring the approach with grasses, structural and functional genomics is also being used to identify and characterize genetic determinants of abiotic stress tolerance in indigenous representatives of the Fabaceae. Tolerance to salt stress is being investigated in the endemic, monospecific *Viminaria juncea* (golden spray) (Figure 1B). Because of a preference for growth in swampy locations, *V. juncea*, like the *Lachnagrostis* spp., also has significant tolerance of waterlogging. *V. juncea* can survive in growth media containing 160 mM NaCl and at 120 mM NaCl maintains dry matter production in 72% of untreated controls. Another indigenous genus of the Fabaceae, *Swainsona*, also includes species with appreciable tolerance of salt stress. Both *S. swainsonoides* (downy Swainson pea) (Figure 1C) and *S. procumbens* (Broughton pea) (Figure 1D) are found growing around the margins of saline lakes in arid areas of southern Australia, whilst *S. lessertifolia* (coast Swainson pea) inhabits coastal sand dunes. In addition, *Glycyrrhiza acanthocarpa* (southern liquorice) has been targeted because it is highly adapted to growth on acidic soils, and to tolerate both the consequent increased availability of $Al^{3+}$ and reduced availability of phosphate.

### EST discovery

To overcome the constraints caused by the large size of plant genomes and the significant proportion of non-coding genomic sequences, an EST-based approach to obtaining sequence information from species targeted in the xenogenomics program has been chosen. EST sequencing is a simple, economical way of accessing the ca. 0.1–10% of the genome that is expressed in target plant organs. In addition, the cDNA clones or their sequences provide a primary resource for microarray-based profiling of mRNA abundance.

To enrich for genes whose products confer tolerance to specific or general abiotic stress, cDNA libraries have been constructed from mRNA derived from selected tissues from stress-treated plants, in comparison to unstressed control samples. It is anticipated that these collections of cDNAs and ESTs will include genes which are transcriptionally stress-induced, some of which are predicted to function in tolerance mechanisms. Care has also been taken to apply the particular stress in a manner that reflects the ecophysiology of the target plant species and an understanding of resident environmental conditions. For example, for the *Lachnagrostis* spp. and *V. juncea*, roots and leaves have been sampled from long-term adapted plants that had salt stress imposed incrementally. In the case of *D. antarctica,* ESTs were derived from root and shoot samples of plants subjected to freezing stress (−16 °C), cold acclimation (5 °C), elevated temperature (25 °C) and to transitions between these temperatures. For *M. stipoides* and *G. acanthocarpa,* only whole root and root tip material was sampled from time courses following aluminium-stress imposition.

Randomly selected, oligo dT-primed, plasmid-borne cDNA clones were subject to 5′-single-pass sequencing. Sequence files were imported into in-house databases, trimmed for vector and low quality sequence, and sequences <100 nt in length were excluded. Table 1 shows the number of high quality ESTs obtained for each species to date and the numbers of unigenes following D2 and cap3 clustering and assembly. The results of sequence similarity searches have been used to assign a putative function to each EST (Figure 2).

### cDNA microarray-based transcript profiling

Microarrays are a powerful method for the global analysis of steady-state intracellular mRNA levels, and thus identifying genes that are transcriptionally modulated as a consequence of metabolic or bioenergetic demands or biotic or abiotic stresses. In

**Table 1.** Gene discovery in indigenous Australian flora and native Antarctic plants

| Organism | ESTs | Unigenes[a] | Novel genes[b] |
|---|---|---|---|
| *Deschampsia Antarctica* | 12 403 | 7736 | 3350 |
| *Lachnagrostis robusta* | 5379 | 2723 | 716 |
| *Lachnagrostis adamsonii* | 4446 | 1730 | 438 |
| *Microlaena stipoides* | 4210 | 2736 | 441 |
| *Viminaria juncea* | 6123 | 4453 | 1726 |
| *Glycyrrhiza acanthocarpa* | 6570 | 4872 | 2202 |

[a] ESTs with >95% identity over >100 nt were clustered.
[b] Unigenes producing a match with probability of $<1 \times e^{-5}$ when used as query for BLASTn search against complete GenBank database.

Copyright © 2005 John Wiley & Sons, Ltd. 

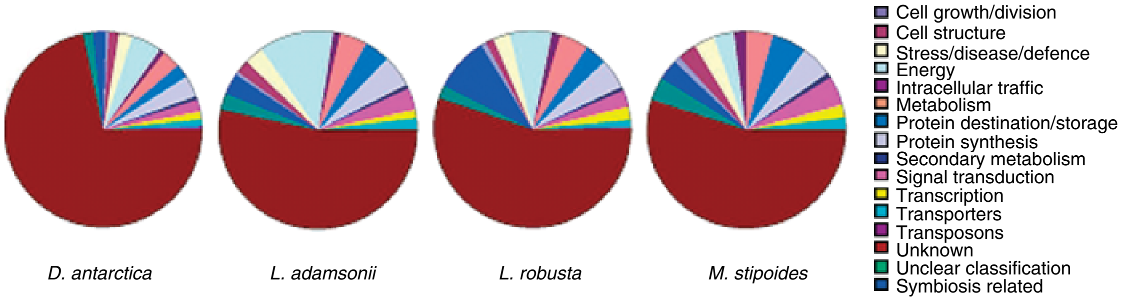

**Figure 2.** Distribution of functional categories amongst EST clusters in xenogenomics target species the context of the substantial proportion of cDNA clones in the collections from each xenogenomics program species, for which primary sequence annotations provide little or no functional insights, microarrays offer the prospect of identifying novel genes implicated in plant responses to abiotic stress. A number of species- and genus-specific spotted microarrays have been designed, fabricated based on PCR amplicons of unigene collections, and used for transcriptome analysis. For example, the *M. stipoides* unigene chip has been interrogated with samples derived from the roots of seedling plants exposed for varying periods to neutral conditions, to media at pH 4.0, and to acidic media containing 1 mM $Al^{3+}$. By applying a complete loop design, it has been possible to distinguish between genes whose transcript levels are elevated in response to low pH, or specifically to aluminium stress.

EST sequencing itself also affords a crude form of profiling transcript levels in the form of an '*in silico* northern analysis'. Frequency of occurrence of ESTs encoding particular products reflects the abundance of the corresponding mRNA in the tissue or treatment being sampled. For example, 0.58% of all ESTs in *M. stipoides* roots exposed to aluminium stress encode homologues of malate dehydrogenase (MDH), compared to 0.1% or less for ESTs from the roots of other grass species. Application of northern hybridization analysis to validate this result has revealed that mRNAs encoding the predominant cytoplasmic form of MDH are no more abundant in response to aluminium stress. However, relative to the mature part of the root and to leaves, transcript levels of this MDH isoform encoding gene are enriched ca. four-fold in root tips, the part of the root that first encounters aluminium and also the part containing the cells most vulnerable to aluminium toxicity.

In addition to coding regions capable of conferring tolerance to abiotic stress, regulatory sequences that are abiotic stress-responsive and/or tissue-specific have been isolated and characterized. Transcript profiling methods such as microarrays enable the identification of genes of both known and unknown function with desirable expression patterns. Adaptor-PCR has been used to isolate 5′-regulatory sequences from a number of genes, including that of the root tip-enriched MDH isoform from *M. stipoides*.

### Candidate gene selection and functional screening

As a result of sequence and transcriptomic analyses, candidates for novel genes or gene variants that are capable of conferring tolerance to abiotic stress have been selected for functional analysis. The criteria that can be applied to identify candidate genes include: putative orthology to genes with demonstrated or inferred involvement in stress tolerance; presence of particular diagnostic protein sequence motifs; or transcript level modulation in response to abiotic stress. Examples of target classes of candidate genes include those involved in transcriptional regulation and signal transduction, genes encoding putative molecular transporters and enzymes involved in osmoprotectant synthesis, genes that are transcriptionally activated in response to cold acclimation, and genes encoding putative antifreeze proteins (AFPs).

A number of different systems and assays have been used for functional screening of candidate genes, including *in planta* screening of candidate genes for desirable phenotypes that result from gain-of-function expression in *Arabidopsis*

Copyright © 2005 John Wiley & Sons, Ltd. 

*thaliana,* followed by visual screens based on root growth established for tolerance of salinity, osmotic and aluminium stresses, using tolerant and sensitive *Arabidopsis* mutant lines to determine conditions under which phenotypic differences are revealed.

Other *in planta* systems are also available for functional analysis. These have the advantages of being closely related to the species from which the candidate genes originated, providing higher levels of biochemical and physiological compatibility and, unlike *Arabidopsis*, are species of agronomic importance. In the Poaceae, these include *Lolium perenne* (perennial ryegrass), *Triticum aestivum* (wheat) and *Oryza sativa* (rice), and in the Fabaceae, *Trifolium repens* (white clover). This taxonomic affinity to the xenogenomics target species also provides the possibility of analysing candidate genes through loss-of-function approaches, using either anti-sense or post-transcriptional gene silencing technologies, providing orthologous sequences can be identified in their genomes.

The experimental strategy and potential for novel allele discovery of the xenogenomics approach has been particularly well exemplified through the identification and functional characterization of a gene product implicated in abiotic stress tolerance, as a contributing determinant to the molecular basis of freezing tolerance in *D. antarctica* [John UP *et al*., unpublished]. We have exploited a simple biophysical assay [7] to establish that *D. antarctica* has potent recrystallization inhibition (RI) activity that prevents small ice crystals becoming consolidated into potentially plasmolytic large ice crystals. Furthermore, this RI activity is highly induced by cold acclimation, is found in the apoplastic (extracellular) spaces where freezing occurs, and is heat soluble (Figure 3). The latter characteristic is diagnostic of an ice recrystallization inhibition protein (IRIP) isolated as an incomplete protein from *L. perenne* [6].

Through examination of our EST collection and by genomic adaptor-PCR and Southern hybridization analysis, *D. antarctica* has been demonstrated to contain a small family of IRIP-encoding genes. Full-length clones of *IRIP* genes from *D. antarctica* encode an apoplastically-targeted product with two distinct domains, each comprised of repeat motifs, the leucine-rich repeat (LRR) and the IRIP repeat, respectively. IRIP orthologues are also found in other Poaceae species but vary considerably in their number of LRRs, from as many as nine to as few as one in the *D. antarctica* forms. Structural modelling of IRIPs has shown that both repeat domains are predicted to adopt structures that are lattice-matched to ice crystal faces. However, minimization of the number of LRR repeats in the *D. antarctica* IRIPs derives a structure predicted to bind with greater affinity to ice, and thus function more efficiently as an IRIP than those from other Poaceae. Transcript profiling using northern hybridization analysis and semi-quantitative reverse transcriptase PCR shows that IRIP transcript levels that are undetectable in plants grown at 25 °C are greatly escalated in response to cold acclimation. Finally, we have exploited a different *in vivo* system for functional analysis to show that *D. antarctica* IRIPs expressed in *E. coli* possess RI activity when assayed.

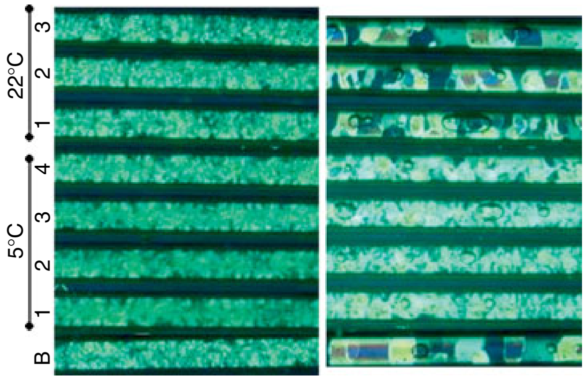

**Figure 3.** Recrystallization inhibition assay on leaf extracts from non-acclimated (grown at 22 °C) and cold-acclimated (5 °C) *D. antarctica*. Upper panel, initial ice crystal structure following snap freezing; lower panel, ice crystal structure after 16 h incubation at −3 °C. Capillary B contains 1000 μg/ml bovine serum albumin; capillaries 1–4: 20, 5, 1.25, and 0.31 μg/ml, respectively, of apoplastic protein

Thus the activity of IRIPs in *D. antarctica* can coherently account for the cryotolerance of this species, offering the prospect of being able to enhance this capacity in economically important crop and pasture plants, and illustrating the utility of the xenogenomics approach for identifying novel sources of genetic diversity.

### Acknowledgements

We thank former and present members of the Xenogenomics Laboratory and our collaborators for their contribution to the work described here. This work was funded by the Australian Research Council, the Grains Research and

Copyright © 2005 John Wiley & Sons, Ltd. 

Development Corporation, the Victorian Department of Innovation, Industry and Regional Development and the Victorian Department of Primary Industries, Australia.

### References

1. Bravo LA, Ulloa N, Zuniga GE, *et al*. 2001. Cold resistance in Antarctic angiosperms. *Physiol Plant* **111**: 55–65.
2. Crawford SA, Wilkens S. 1998. Effect of aluminium on root elongation in two Australian perennial grasses. *Aust J Plant Physiol* **25**: 165–171.
3. Kore-eda S, Cushman MA, Akselrod I, *et al*. 2004. Transcript profiling of salinity stress responses by large-scale expressed sequence tag analysis in *Mesembryanthemum crystallinum*. *Gene* **341**: 83–92.
4. Mehta PA, Sivaprakash K, Parani M, *et al*. 2004. Generation and analysis of expressed sequence tags from the tolerant mangrove species *A. vicennia marina* (Forsk) Vierh. *Theor Appl Genet* (in press).
5. Rudd S. 2005. OpenSputnik — a database to ESTablish comparative plant genomics using unsaturated sequence collections. *Nucleic Acids Res* **33**(database issue): D622–627.
6. Sidebottom C, Buckley S, Pudney P, *et al*. 2000. Heat-stable antifreeze protein from grass. *Nature* **406**: 256.
7. Tomczak MM, Marshall CB, Gilbert JA, *et al*. 2003. A facile method for determining ice recrystallization inhibition by antifreeze proteins. *Biochem Biophys Res Commun* **311**(4): 1041–1046.

Copyright © 2005 John Wiley & Sons, Ltd. 
